# Case report: GCA like picture-preceding inaugural MOGAD presentation: A patient with a sudden-onset uniocular blindness

**DOI:** 10.1097/MD.0000000000036326

**Published:** 2023-12-08

**Authors:** Yixuan Zeng, Xuan Liu, Runtao Bai, Yanxia Zhou, Lijie Ren

**Affiliations:** a Department of Neurology, The First Affiliated Hospital of Shenzhen University, Shenzhen Second People’s Hospital, Shenzhen, China; b Department of Gerontology, Shangrao People’s Hospital, Shangrao, China.

**Keywords:** autoimmune, giant cell arteritis, MOG-IgG-associated disease, myelin oligodendrocyte glycoprotein

## Abstract

**Rationale:**

Myelin oligodendrocyte glycoprotein antibody-associated disorders (MOGAD) represents a demyelinating neurological syndrome characterized by the presence of serum IgG antibodies directed against myelin oligodendrocyte glycoprotein (MOG-IgG). Concurrently, giant cell arteritis (GCA) constitutes a systemic autoimmune vasculitis.

**Patient concerns:**

In this case, we describe an elderly female patient who presented with the sudden onset of a severe headache, unilateral blindness, and clinical manifestations resembling those of GCA.

**Diagnosis:**

Upon conducting a comprehensive analysis of serum antibodies, the diagnosis of MOGAD was established due to the presence of detectable serum MOG-IgG.

**Interventions:**

Subsequently, the patient was administered intravenous methylprednisolone therapy, commencing 27 days after the initial onset of symptoms.

**Outcomes:**

It is noteworthy that patients afflicted by MOGAD typically manifest severe visual impairment, which, in many instances, exhibits significant improvement following immunotherapeutic interventions. However, this particular patient did not experience any amelioration in visual function despite glucocorticoid therapy.

**Lessons:**

This unique case illustrates that the clinical presentation resembling GCA may precede the inaugural manifestation of MOGAD. This suggests the possibility of immune-mediated arterial involvement. The significance of glucocorticoid therapy in the context of immune-related diseases warrants further scrutiny, particularly in cases where MOG-IgG screening should be promptly considered.

## 1. Introduction

As a form of vasculitis, temporal arteritis is also known as giant cell arteritis (GCA). GCA is a chronic, idiopathic, granulomatous vasculitis. GCA is one of the most common vascular disorders, usually affecting patients over 50 years of age. New-onset headache, scalp tenderness, visual disturbances, and jaw claudication are all common clinical features of GCA.^[1]^ GCA causes irreversible and rapid vision loss in the elderly. Numerous cases have reported that ischemic stroke is often complicated with GCA.^[2,3]^ Myelin oligodendrocyte glycoprotein antibody-associated disorders (MOGAD) has been of significant concern as a cause of inflammatory demyelinating disease of the central nervous system over the past years.^[2,4]^ Myelin oligodendrocyte glycoprotein (MOG) regulates the stability of oligodendrocyte microtubules, maintaining the structural integrity of the myelin sheath through its adhesion properties, as well as mediating the interactions between myelin and the immune system. Compared with other patients with aquaporin 4 positive neuromyelitis optica spectrum disorder, MOGAD patients are often accompanied by optic disc edema. MOGAD patients always present with severe visual impairment, and a better vision recovery prognosis than aquaporin 4 positive neuromyelitis optica spectrum disorder. Herein, we report a woman with an initial presentation of headache with sudden uniocular visual loss and a clinical diagnosis of GCA. But final antibody test led to a possible diagnosis of MOGAD. Both MOGAD and GCA are immune-mediated diseases. But by now, there’s no reported cases of GCA with positive antibodies against myelin oligodendrocyte glycoprotein (MOG-IgG) or MOGAD patients with GCA-like presentation. Considering the unique clinical manifestations of this patient, this case is reported as follows, in order to improve the understanding of clinicians for both MOGAD and GCA.

## 2. Case presentation

**Figure1.**
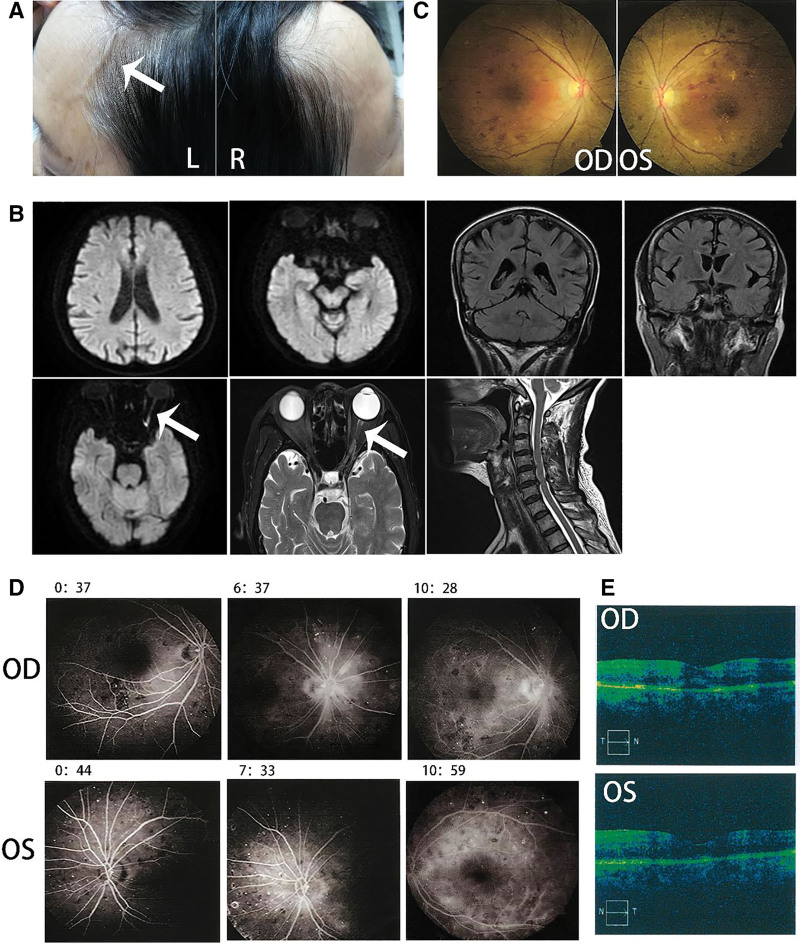
(A) Prominence of the left temporal artery (white arrow) while the right temporal artery is normal. (B) Hyperintensities of the left optic nerve via MRI (white arrow). No remarkable finding within the spinal cord upon MRI. (C) Fundus photography showed pale binocular disc and miniature blood vessels in both eyes. (D) Fundus fluorescein angiography showed clusters of high fluorescence penetration and scattered patchy fluorescence shielding in both of the optic discs. (E) OCT showed the retinal nerve fiber layer around the left optic disc was significantly thinner. MRI = magnetic resonance imaging, OCT = optical coherence tomography.

**Figure 2.**
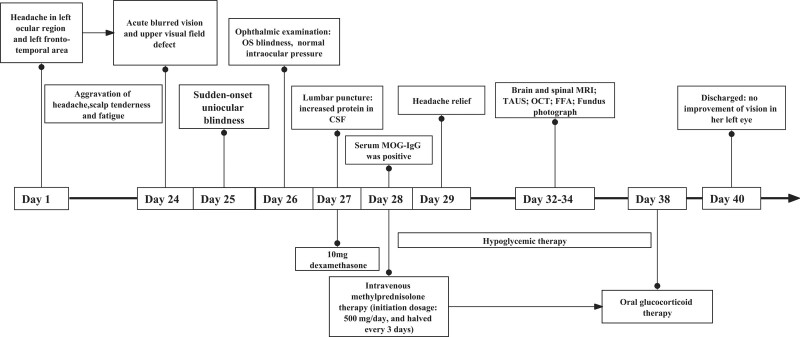
The schema of the clinical manifestations and management of our patient.

A 61-year-old woman experienced an acute paroxysmal throbbing pain in the left ocular region that then spread to the left fronto-temporal area without any noticeable aura (day 1). At the beginning, headaches were mild and did not affect her daily activities, with a frequency of 1 to 3 headaches per day. The headaches were accompanied by scalp tenderness that can be relieved by analgesics or rest. About a week later, the headaches become more and more frequent and eventually lasting all day. She then reported a complaint of fatigue as the headaches interrupted her daily sleep. On day 25, she suddenly developed acute blurred vision and upper visual field defects in the left eye. Symptoms then progressed to complete vision loss in the left eye within 24 hours, as well as severe vomiting. She was then immediately brought to a local emergency and got a primary diagnosis of ischemic optic neuropathy (AION). Past history tells that she had diabetes and anemia, with daily maintenance medication worked well. An ophthalmic examination revealed her right visual acuity was 0.5 (30/60) based on the Snellen chart and there was no light perception in the left eye. Intraocular pressure was normal (right eye: 10 mmHg, left eye: 9 mmHg) and no typical “cherry red spot” sign nor hemorrhage sign was observed using a portable ophthalmoscope. She was then transferred to our hospital and was hospitalized in the neurology department (day 27). Neurological examination showed tenderness, prominence, and decreased temporal artery pulsation. There was complete blindness and incomplete abduction in the left eye (Fig. 1A). As an elderly woman who presented new-onset localized headache, temporal artery tenderness, decreased pulsation, and uniocular blindness, she got a primary diagnosis of GCA according to the American College of Rheumatology 2021 criteria for GCA.^[5]^ Based on these findings, dexamethasone was used with an initial dosage of 10mg per day. Laboratory results revealed normal elevations of inflammation biomarkers including ESR levels of 11 mm/h and a C-reactive protein level of 0.5 mg/dL (normal range: 0–20 mm/h and 0–5 mg/L, respectively). There was an increase in red blood cells (5.13 × 10^12^/L, reference range: 4.30–5.80 × 10^12^/L) and a decrease in hemoglobin (103 g/L, reference range: 130–175 g/L). Preprandial glucose was out of normal range (8.15 mmol/L, reference range: 3.89–6.11 mmol/L). The antinuclear antibody (1:100, reference range: <1:100) test was positive. Tumor markers (AFP, CEA, CA199, CA125, CA153, and ferritin), electrolytes, renal function, liver function, syphilis, and HIV tests were all unremarkable. A lumbar puncture was performed immediately after hospitalization. The cerebrospinal fluid showed increased protein levels (691.1 mg/L, reference range:150–450 mg/L). While the leukocyte, chloride, and glucose results were all unremarkable. Based on previous findings, we further tested antibodies targeting optic neuritis (ON) (day 28). Cell-based assays (CBA) revealed that serum MOG-IgG was positive with a low titer of 1:10. The oligoclonal bands result in cerebrospinal fluid was normal. Based on the diagnosis of MOGAD, she then received intravenous methylprednisolone therapy (initiation dosage: 500 mg/day and halved every 3 days) (from day 28). Temporal artery ultrasonography was performed on day 32 and no abnormality was found. Furthermore, a hyperintensity of the left optic nerve was found in magnetic resonance imaging (MRI), without abnormality in spinal cord MRI (Fig. 1B). At the same time, no “cherry red spot” nor signs of hemorrhage sign were found in the fundus camera during examination (Fig. 1C). Fundus fluorescein angiography revealed diabetic retinopathy (Fig. 1D). Optical coherence tomography showed a thinning of the retinal nerve fiber layer around the left optic disc (Fig. 1E) (day 33–34). The patient refused an arterial biopsy further. Her headache was relieved significantly after methylprednisolone treatment. Unfortunately, there was no significant improvement of vision in her left eye (the case summary in Fig. 2). She then received a continuation of the glucocorticoids tapered dose for a period of 6 months. During the 2-year follow-up period, she did not experience any recurrence of ON or headache, nor any sign of new-onset of other rheumatic diseases, such as polymyalgia rheumatica, or Takayasu arteritis.

## 3. Discussion

In summary, this case report details an elderly female patient who presented with a sudden-onset severe headache, unilateral blindness, and clinical features reminiscent of GCA. However, a thorough analysis of serum antibodies led to the definitive diagnosis of MOGAD due to the presence of serum MOG-IgG. What sets this case apart is the lack of visual improvement following glucocorticoid therapy, a departure from the typical response seen in MOGAD patients. This intriguing observation hints at the potential immune-mediated involvement of arteries in MOGAD and underscores the need for a deeper understanding of the interplay between these autoimmune conditions. It also raises questions about the optimal management of such dual-diagnosis scenarios, shedding light on the importance of timely MOG-IgG screening in selected cases with clinical presentations resembling GCA, with far-reaching implications for patient care and future research endeavors.

It is unclear what mechanism of action drives GCA-like presentation as we didn’t get a temporal artery biopsy result.^[3,6]^ GCA was thought to be caused by the immune system’s abnormal attacks on arteries. GCA often happens in females over 50. A history of polymyalgia rheumatica or other auto-immune associated diseases will increase the risk of GCA. The neuropathy of GCA is arteritis ischemic optic disease. And corticosteroid drug is the main treatment for GCA. Headaches, scalp tenderness, vision problem and sudden blindness, were all typical symptoms of GCA in this patient, lead to the dexamethasone treatment for the rescue of her vision. However, fundus camera result of this patient reveals that no sign of AION. Furthermore, in MOGAD patients, ON often happens bilaterally, and have a relatively better prognosis. Post-bulbar pain is also common in MOGAD patients, indicating retrobulbar ON. For this patient, whether this is a comorbidity of GCA with MOGAD or just a rare GCA manifestation of MOGAD is not yet known. Additionally, for both GCA and MOGAD, high dose of corticosteroid is the first-line therapeutic recommendation. The pathophysiological effects of the possible coexistence of MOGAD and GCA have yet to be elucidated.

Typical clinical sign of MOGAD include bilateral ON, myelitis, brain encephalitis, and encephalitis. The clinical features of disease differ depending on the onset-age. The most common manifestations in adults NMO patients are simultaneous occurrence of ON and myelitis. However, sudden-onset uniocular ON with temporal artery swelling were found in our case. One hypothesis is that MOGAD may also be accompanied by temporal artery abnormalities. The specific physical examination of the temporal artery is only a rare symptom of MOGAD since inflammation within the intra-orbital and peri-optic regions are also involved in MOGAD.^[7]^ MOGAD patients primarily have headaches as well as neck and neuropathic pain. Previous research has reported that approximately half of the MOGAD patients have prodromal headaches.^[8,9]^ Headaches usually start a few days before visual defects and extend from the ocular region to the fronto-temporal area.^[10]^ Lab examinations in this case revealed that the reason for uniocular blindness is ON rather than AION. And combined with the MRI results of the optic nerve involved, we can tell the diagnosis of this patient is more MOGAD than simply GCA.

Further, even though we did not get available biopsy evidence, the signs of scalp tenderness and temporal artery swelling still indicate arteritis. The coexistence of the temporal arteritis may come from a faulty “attack” to vessel wall triggered by an immune response. GCA is characterized by segmental and focal panarteritis with non-necrotizing granulomatous inflammation.^[1]^ And it is characterized by arterial wall infiltration by T lymphocytes, macrophages, and multinucleated giant cells.^[11]^ It is always accompanied by numerous autoimmune diseases, such as autoimmune encephalitis.^[12,13]^ The predominant subtype of IgG1 antibodies reactive in MOG initiates the central nervous system demyelinating process via T cell-mediated cytotoxicity and B cell-mediated immune responses with complement activation.^[14,15]^ Pathogenesis studies of MOGAD can be characterized by perivascular infiltrated MOG-laden macrophages and CD4 + T-cell infiltration, suggesting that MOGAD can activate macrophages and produce blood vessel-related inflammation. Conversely, although the mechanisms responsible for the coexistence of double or more autoantibodies are still undetermined. This phenomenon may be partially explained by the idea of epitope spreading^[16]^; thus, the persistent recognition and activation of self-antigens leads to chronic immune responses associated with the development of antibodies against diverse dominant epitopes within the same antigen (intramolecular) or to different antigens (intermolecular). As MOGAD and GCA are both inflammatory diseases, the subsequent immunotherapy is equally effective. In our case, the sequence of onset or the mechanism of interaction between GCA and MOGAD is still unclear as there was no biopsy evidence. However, more research is needed to explain the coexistence of GCA and MOGAD.

It is imperative to acknowledge that a crucial limitation of this study resides in the patient’s non-consent for biopsy, a procedure of paramount importance for definitively confirming the diagnosis of giant cell arteritis (GCA) by elucidating its distinctive histopathological features within inflamed arterial tissues. The absence of a confirmatory biopsy in this instance signifies a notable constraint, impeding the unequivocal establishment of GCA as a concurrent comorbid condition. Consequently, while clinical manifestations exhibited a parallelism to GCA, the absolute exclusion of this diagnosis remained unattainable. This limitation serves as a poignant reminder of the diagnostic intricacies encountered in cases where invasive diagnostic measures are precluded, whether due to patient inclinations or clinical constraints. Furthermore, it should be noted that convincing patients to undergo biopsy, despite its designation as the recommended initial diagnostic procedure,^[17,18]^ can be a formidable challenge. In light of our case’s pivotal reliance on disease-specific antibodies, the exploration of antibody screening in the context of demyelinating neurological disorders is warranted, particularly among patients with GCA-like presentations, especially in the absence of classical ophthalmic evidence. While glucocorticoids stand as the preferred initial therapeutic approach for both GCA and MOGAD, a comprehensive evaluation of therapeutic options is indispensable, particularly when confronted with the co-occurrence of these 2 distinct maladies.^[19,20]^ It is worth underscoring that the intricate relationship between MOGAD and GCA remains shrouded in uncertainty. Consequently, the imperative for more expansive, large-scale investigations is incontestable, with the objective of elucidating the robustness of this association. Furthermore, vigilance should be exercised in cases where patients present with symptoms of headache and ON, warranting comprehensive assessment and a multidiscipline.

## Acknowledgments

The authors would like to thank the patient and her family for their participation and help.

## Author contributions

**Conceptualization:** Lijie Ren.

**Data curation:** Xuan Liu.

**Formal analysis:** Yanxia Zhou.

**Funding acquisition:** Yixuan Zeng.

**Investigation:** Yixuan Zeng, Xuan Liu.

**Supervision:** Runtao Bai, Yanxia Zhou.

**Validation:** Runtao Bai, Lijie Ren.

**Visualization:** Lijie Ren.

**Writing – original draft:** Yixuan Zeng.

**Writing – review & editing:** Yixuan Zeng.
